# An Improved Image Classification Method for Cervical Precancerous Lesions Based on ShuffleNet

**DOI:** 10.1155/2022/9675628

**Published:** 2022-09-13

**Authors:** Shan Fang, Jiahui Yang, Minghui Wang, Chunhui Liu, Shuang Liu

**Affiliations:** ^1^College of Quality and Technical Supervision, Hebei University, Baoding 071002, China; ^2^Affiliated Hospital of Hebei University, Baoding 071002, China

## Abstract

With the rapid development of deep learning, automatic lesion detection is used widely in clinical screening. To solve the problem that existing deep learning-based cervical precancerous lesion detection algorithms cannot meet high classification accuracy and fast running speed at the same time, a ShuffleNet-based cervical precancerous lesion classification method is proposed. By adding channel attention to the ShuffleNet, the network performance is improved. In this study, the image dataset is classified into five categories: normal, cervical cancer, LSIL (CIN1), HSIL (CIN2/CIN3), and cervical neoplasm. The colposcopy images are expanded to solve the problems of the lack of colposcopy images and the uneven distribution of images from each category. For the test dataset, the accuracy of the proposed CNN models is 81.23% and 81.38%. Our classifier achieved an AUC score of 0.99. The experimental results show that the colposcopy image classification network based on artificial intelligence has good performance in classification accuracy and model size, and it has high clinical applicability.

## 1. Introduction

Cervical cancer is the fourth most common female cancer. The statistics of WHO show roughly 604,000 new cases worldwide in 2020, accounting for 6.5% of all new cancer cases in women [1]. The early cure rate of cervical cancer is high, but the lack of signs and symptoms at this stage hinders the early diagnosis. A successful screening program can avoid cervical cancer death and reduce the incidence and persistence of the disease [2]. According to statistics, more than 311,000 cervical cancer deaths occur every year. Due to the lack of experienced health care staff and insufficient funds for the screening system, cervical cancer screening facilities are very scarce in developing countries [3]. Therefore, it is necessary to use automated and effective screening methods, to reduce the cost of early detection of cervical cancer. Cervical cancer screening follows the following workflow: HPV test, cytology or PAP smear test, colposcopy, and biopsy [4]. The PAP smear image screening is to take a small number of cell samples from the cervix of the uterus, placing them on glass slides, and then study whether they are abnormal under a microscope. This method is time-consuming and depends on the experience of pathologists. Different pathologists will see different results in the same film. The HPV test is a DNA test. PAP smear and HPV test are very expensive treatments with low sensitivity. Therefore, colposcopy is widely used in developing countries. Colposcopy identifies cervical lesions by using a low magnification microscope under a strong light source [5]. Its accuracy highly depends on the skills of physicians. There are significant differences in the detection rate of lesions among different colposcopy physicians. This has aroused people's attention to the insufficient diagnosis of lesions (including missed diagnosis of cervical cancer) and excessive diagnosis of lesions [6–9]. Excessive diagnosis of lesions may lead to the excessive treatment of low-grade cervical lesions, increasing the risk of infection and economic burden [10].

In recent years, deep learning has gradually become popular in the field of medicine. The purpose of medical image processing is to restore the original unclear image, to highlight some characteristic information in the image, or to classify the image. Medical images include MRI, CT, ultrasound images, and blood smear images [11, 12]. Convolutional neural network (CNN) is an important end-to-end deep learning model [13], which is mainly used in image recognition, segmentation, and target detection in medical image processing. Ai-assisted colposcopy can help colposcopy specialists improve their diagnostic performance, optimize clinical workflow, and relieve pressure on colposcopy physicians and hospitals, which has great potential to improve cervical cancer screening performance.

We propose a method for the classification of cervical precancerous lesions based on deep learning. The main contributions of this paper are as follows:

- Different grades of cervical precancerous lesions, cervical neoplasm, and cervical cancer were classified.
- A deep inverted residual network based on the improved additional channel attention of ShuffleNet is proposed.
- Compared with the traditional residual network, the inverted residual network can not only ensure the automatic extraction of features in the image but also reduce the amount of calculation and improve the calculation speed of the model.

The structure of this paper is as follows: Section 2 introduces the proposed deep learning model.  Section 3 describes data sources and processing.  Section 4 is experiment and analysis.  Section 5 concludes this work.

## 2. Materials and Methods

### 2.1. Depthwise Separable Convolution

Deep CNN networks such as ResNet [14] and DenseNet [15] have greatly improved the accuracy of image classification. However, in addition to accuracy, computational complexity is also an important index to be considered by the CNN network. Complex networks may run slowly. Some specific scenes, such as an unmanned vehicle, need low latency, and edge computing devices also need small models that are both accurate and fast. To meet this demand, lightweight deep learning networks such as MobileNet [16] and ShuffleNet [17] have been proposed, which achieve a good balance between speed and accuracy.

To speed up the calculation speed of the network and reduce the amount of calculation, MobileNet proposes depthwise separable convolution. For the traditional convolution, an input feature graph with a size of (*W*, *H*, *C*_in_) is used to obtain an output feature graph with a size of (*W*, *H*, *C*_out_) through convolution operation using an *N* × *N* convolution kernel. At this point, the computational quantity is

(1) $$\begin{array}{c} W\times H\times C_{\text{in}}\times C_{\text{out}}\times N\times N. \end{array}$$

Depthwise separable convolutions are divided into depthwise convolutions and pointwise convolutions. Depthwise convolutions are equivalent to using the convolution kernel with the number of channels of 1 to perform separate convolution operations on each channel of the input feature map. The feature map with the same number of output and input channels needs to be multiplied *W* × *H* × *C*_in_ × *N* × *N* times. Pointwise convolution, a simple 1 × 1 convolution, needs *W* × *H* × *C*_in_ × *C*_out_ times of multiplication calculation. Compared to ordinary convolution, the calculation amount of depthwise separable convolution can be reduced:

(2) $$\begin{array}{c} \frac{W\times H\times C_{\text{in}}\times N\times N+W\times H\times C_{\text{in}}\times C_{\text{out}}}{W\times H\times C_{\text{in}}\times C_{\text{out}}\times N\times N}=\frac{1}{C_{\text{out}}}+\frac{1}{N\times N}. \end{array}$$

### 2.2. Inverted Residual Network with Additional Channel Attention

**Figure 1:**
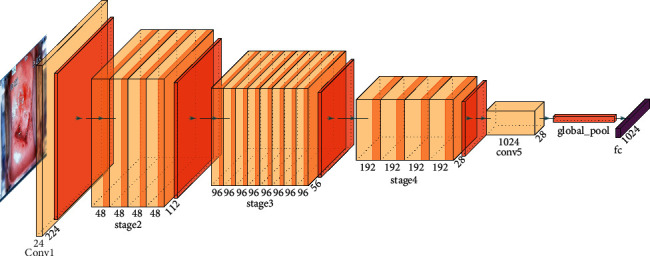
ShuffleNet structure model.

**Figure 2:**
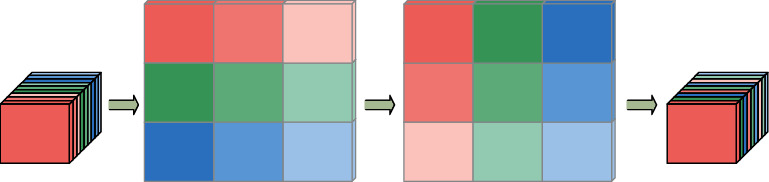
Channel shuffle process.

ShuffleNet has similar ideas with MobileNet, Xception [18], and ResNet. It uses channel shuffle and depthwise separable convolution to optimize the residual structure of ResNet, which not only ensures the network accuracy but also improves the operational efficiency of the model. Unlike the traditional residual module, which directly integrates the features of the deep networks and nondeep networks obtained through multiple convolutions, the inverted residual module divides the input feature map into two batches X1 and X2, X2 through depthwise separable convolution and twice 1 × 1 convolution + batch standardization + activation function, X1 and X2 are fused with deep and nondeep features, and finally, channel shuffle is used to mix deep and nondeep features. Suppose that the input layer is divided into *G* groups, and the total number of channels is *G* × *n*. First, divide the channel into two dimensions (*G*, *n*), then transpose these two dimensions into (*n*, *G*), and finally reshape them into one dimension *G* × *n*. The ShuffleNet structure model is shown in Figure 1. The channel shuffle process is shown in Figure 2.

**Figure 3:**
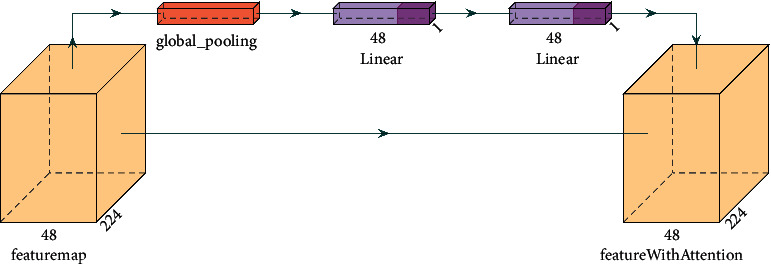
Squeeze-and-Excitation networks structure model.

To make the classification more accurate, we add the Squeeze-and-Excitation Networks (SE) [19] and the Selective Kernel Networks (SK) [20] to the model, respectively. The SE model is shown in Figure 3. Firstly, a feature map U with a total number of channels C and a size of *H* × *W* is flattened into a feature vector of (1, 1, *C*) by a global pooled Fsq shown as follows:

(3) $$\begin{array}{c} Z_{c}=F_{sq}\left(U_{c}\right)=\frac{1}{H\times W}\sum_{i=1}^{H}\sum_{j=1}^{W}U_{c}\left(I,j\right). \end{array}$$

The activation function and linear mapping are added to the feature vector to add more nonlinear conditions, which can better fit the complex correlation between channels. Finally, the calculated channel features are multiplied by the original feature map to obtain the output of channel attention. The SE model strengthens the important features and weakens the unimportant features by controlling the size of the channel proportion, to make the extracted features more directional.

**Figure 4:**
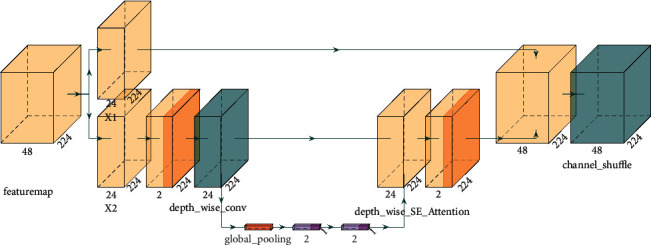
Inverted-residual-SE structure model.

Channel attention is allowed to be inserted between each feature map. After the SE channel attention is inserted into the depth-separable convolution, feature extraction of channel dimension is carried out on the depthwise separable convolution output. The inverted residual network structure model fusing the SE module is shown in Figure 4.

**Figure 5:**
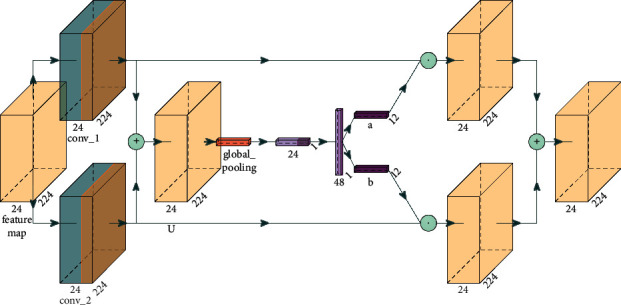
Selective Kernel attention structure model.

SK is mainly the same as SE. The difference is that SENet performs attention on the channel, while SKNet performs attention on the convolution kernel. SKNet uses convolution check feature maps of different sizes in the network to extract features of different scales and then extracts channel attention after fusion of features of different scales. Its network model is shown in Figure 5.

**Figure 6:**
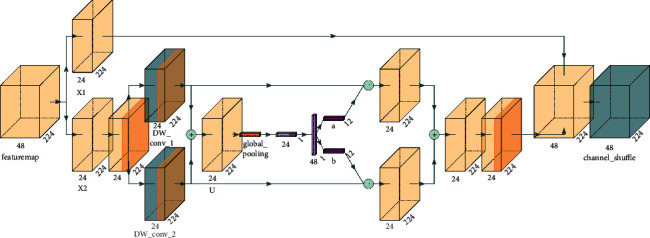
Inverted-residual-SK structure model.

To compare with SE, SK is also used in depthwise separable convolution. The inverted residual network structure model fusing the SK module is shown in Figure 6.

First, the input feature maps are computed by a depthwise separable convolution conv_1 with a convolution kernel size of 3 × 3, and a depthwise separable convolution with a convolution kernel size of 3 × 3 and a dilation factor of 2 in different scales; then the two output feature maps are summed for global pooling, and the pooling layer is computed similar to the SE channel attention; subsequently, the output of two-channel features are multiplied with conv_1 and conv_2 in the channel dimension to obtain two feature maps of mixed channel attention at different scales, and then the two feature maps are summed to obtain the SK attention output features.

## 3. Data Source and Processing

### 3.1. Cervigram Dataset

The cervical cancer screening dataset was provided by the Department of Gynecology, Affiliated Hospital of Hebei University (as show in Table 1). The dataset consisted of colposcopy images of different grades of precancerous lesions (normal, CIN1, and CIN2/3), cervical neoplasm, and cervical cancer. There are 1,189 patients, totaling 6,996 images.

### 3.2. Dataset Making Principle

In this study, data split into training and validation subsets using a 90% to 10% ratio.

Since the uneven distribution of the provided dataset in each category and the number of samples is small, data augmentation is used to add images for five categories (normal, cervical cancer, HSIL, LSIL, and cervical neoplasm). Data augmentation is used to improve the overall structural security of the trained model. There are two ways to enhance the data: one is to get new images; another method is to augment the data, i.e., to create more available data using already available data such as flips, translations, or rotations to make the neural network more generalizable. Three data augmentation methods used in this paper are as follows:

- Randomly cropping the image size to 224 × 224
- Image standardization processing
- Random horizontal and vertical image flipping

## 4. Experiments and Performance Analysis

### 4.1. Experimental Conditions

To ensure the iterative efficiency and improve the model stability and generalization ability, in this study, the network parameters are optimized by stochastic gradient descent (SGD) algorithm using nesterov gradient descent with weight decay of 1*e* – 4, learning momentum of 0.9, and several single batch treatments of 32. Each model is trained for 100 cycles, and the initial learning rate is set to 0.05. The CNN algorithm is implemented in PyTorch coding framework. Model training and evaluation are conducted using Intel (R) Xeon (R) Gold 6240 CPU@2.60 GHz and NVIDIA RTX 2080ti GPU. All programs run on Ubuntu 18.04.5 LTS.

### 4.2. Evaluation Metrics

To evaluate the algorithm effectively, this paper uses training loss and model accuracy for measurement in the training phase. In the test phase, this paper introduces the confusion matrix as the basic evaluation criterion, and the confusion matrix contains four parts of information:

- TN, which is the true negative, can represent the number of negative samples predicted as negative
- TP, which is the true positive, can represent the number of positive samples predicted as positive
- FN, which is the false negative, can represent the number of positive samples predicted as negative
- FP, which is the false positive, can represent the number of negative samples predicted as positive

Since the proposed model is a multiclassification model, accuracy, precision, recall, and F1-scores can be calculated according to the above four indicators. The area-under-the-curve (AUC) score and the confusion matrix are also used to evaluate the performance of the model. The classification accuracy, precision, recall, and F1 score can be obtained by (4)–(7).

(4) $$\begin{array}{c} \text{Accuracy}\left(\text{\%}\right)=\frac{\text{TP}+\text{TN}}{\text{TP}+\text{FP}+\text{TN}+\text{FN}}\times 100, \end{array}$$

(5) $$\begin{array}{c} \text{Precision}\left(\text{\%}\right)=\frac{\text{TP}}{\text{TP}+\text{FP}}\times 100, \end{array}$$

(6) $$\begin{array}{c} \text{Recall}\left(\text{\%}\right)=\frac{\text{TP}}{\text{TP}+\text{FN}}\times 100, \end{array}$$

(7) $$\begin{array}{c} F1-\text{score}\left(\text{\%}\right)=\frac{2\times \text{Recall}\times \text{Precision}}{\text{Recall}+\text{Precision}}\times 100. \end{array}$$

The receiver operating characteristic (ROC) curve is a comprehensive index that shows continuous changes in sensitivity and specificity. According to the position of the curve, the whole graph is divided into two parts. The area under the curve is called AUC. The higher the AUC score, the better the performance of the classification model. The confusion matrix reflects the confusion caused by the classifiers when dealing with multiclassification problems. The value on the diagonal represents the number of correctly classified images of each class. The darker the diagonal color, the better performance of the classifiers. In this paper, the prediction results are normalized.

### 4.3. Contrasting Experimental Results and Analysis

To evaluate the effectiveness of the classification network proposed in this paper, we compare the proposed neural network model with VGG-16 [21], ResNet34, GoogleNet [22], DenseNet121, MobileNet, ShuffleNet, ShuffleNet_SK, and ShuffleNet_SE. To compare the results more confidently, all models use the dataset in this paper and are trained in the same training environment. As shown in Table 2, this study compares the accuracy, precision, recall, and F1-scores of the above networks. The mean and standard deviation were used to summarize the results. The results show that the classification ability of the improved network is significantly improved.

**Figure 7:**
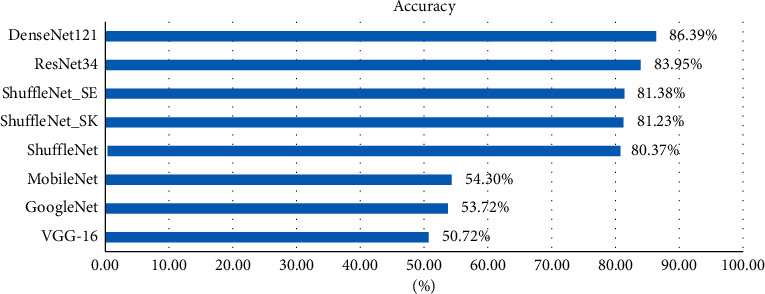
Accuracy comparison.

**Figure 8:**
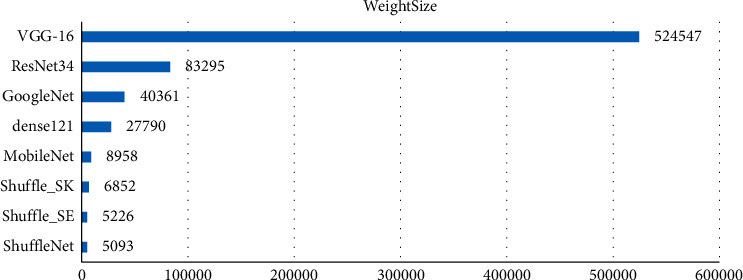
Model size comparison.

Figures 7 and 8 show that the model size of the improved network is greatly reduced compared with the traditional classification network and also greatly reduced compared with the lightweight network MobileNet. In terms of classification accuracy, the improved network maintains high recognition accuracy, and the classification performance is improved compared with the unimproved ShuffleNet and significantly improved compared with MobileNet. Our model can improve computational efficiency significantly while achieving good performance in terms of classification accuracy, thus representing a reasonable balance between model size and performance.

**Figure 9:**
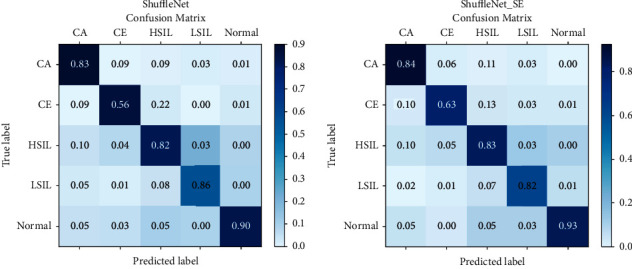
Confusion matrix.

As shown in Figure 9, the improved network may not be as effective as the network before improvement in one index, the prediction accuracy of the network model with SENet added is better.

## 5. Conclusions

This paper has proposed a dataset of colposcopic images using colposcopic images of cervical precancerous lesions and cervical cancer patients of different grades. We have also used six neural network models for comparative experiments and proposed two new deep learning-based lightweight network models ShuffleNet_SK and ShuffleNet_SE for multiclassification of cervical diseases. The classification performance is improved by adding attention on the inverted residual network. As a result, ShuffleNet_SK and ShuffleNet_SE achieved classification accuracy of 81.23% and 81.38%, respectively. The proposed networks are suitable for the mobile terminal with limited computing resources, which can classify cervical diseases more accurately and faster, so as to meet the demand of real-time, and has more practical clinical application value. Additionally, they can also be applied to prescreen for other types of cancer, reducing missed detection by physicians.

## Figures and Tables

**Table 1:** Cervix types in cervigram dataset.

Type	Label	Number of images
Normal	0	2352
LSIL	1	780
HSIL	2	2532
Cervical cancer	3	408
Cervical neoplasm	4	924

**Table 2:** Network comparison experimental data.

Method	Accuracy (%)	Precision (%)	Recall (%)	*F*1-score (%)
VGG-16	50.72 ± 2.12	45.63 ± 3.25	45.67 ± 1.02	45.07 ± 1.59
ResNet34	83.95 ± 4.02	84.88 ± 3.18	81.28 ± 4.51	82.81 ± 3.44
GoogleNet	53.72 ± 5.42	47.43 ± 4.77	51.73 ± 4.82	45.09 ± 5.03
DenseNet121	86.39 ± 1.45	87.00 ± 1.91	83.95 ± 2.62	85.17 ± 1.98
MobileNet	54.30 ± 1.57	65.12 ± 2.18	44.60 ± 1.69	43.45 ± 2.03
ShuffleNet	80.37 ± 2.06	79.90 ± 1.89	79.42 ± 1.58	79.60 ± 1.95
ShuffleNet_SK	81.23 ± 2.03	81.65 ± 1.64	79.88 ± 2.25	80.67 ± 1.83
ShuffleNet_SE	81.38 ± 1.95	81.76 ± 2.32	80.74 ± 1.87	81.16 ± 2.26

## Data Availability

The data are publicly available at https://github.com/AluminiumOxide/ShuffleNet_Attention_Extend.
